# Supplementary figures and images for: Membrane-binding mechanism of the EEA1 FYVE domain revealed by multi-scale molecular dynamics simulations
Source: PLoS Comput Biol. 2021 Sep 23;17(9):e1008807. doi: 10.1371/journal.pcbi.1008807 (PMC8491906; doi:10.1371/journal.pcbi.1008807)

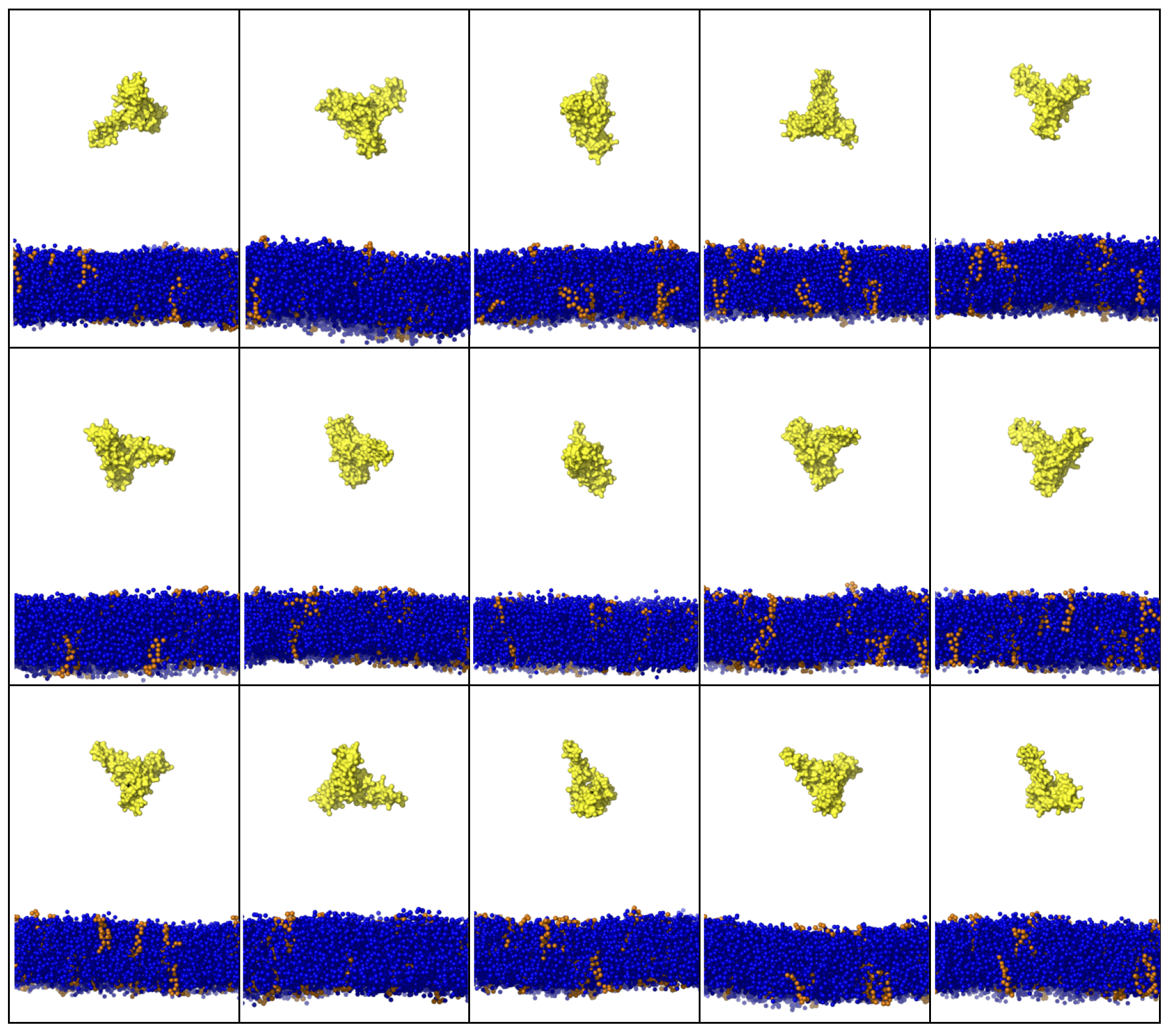

Supplement: S1 Fig — Snapshots from the 15 repeats of the CG-MD, taken after equilibration. FYVE (yellow) is in a random orientation above the membrane composed of POPC (blue) and POPI(3)P (orange). (TIF) [file pcbi.1008807.s001.tif]

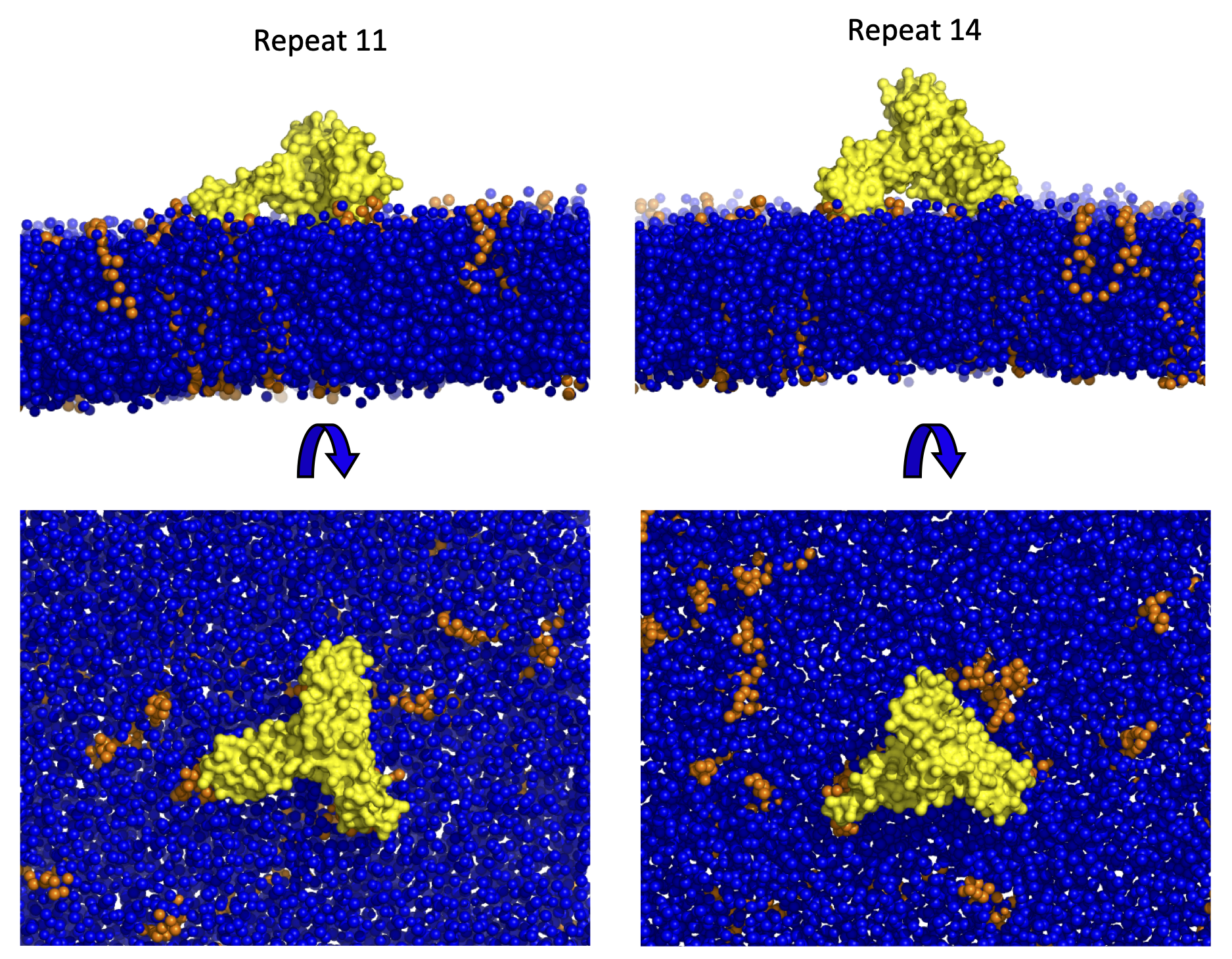

Supplement: S2 Fig — Snapshots of the non-canonical binding modes seen in repeat 11 and repeat 14. FYVE (yellow) bound to the lipid membrane composed of POPC (blue) and POPI(3)P (orange). (TIF) [file pcbi.1008807.s002.tif]

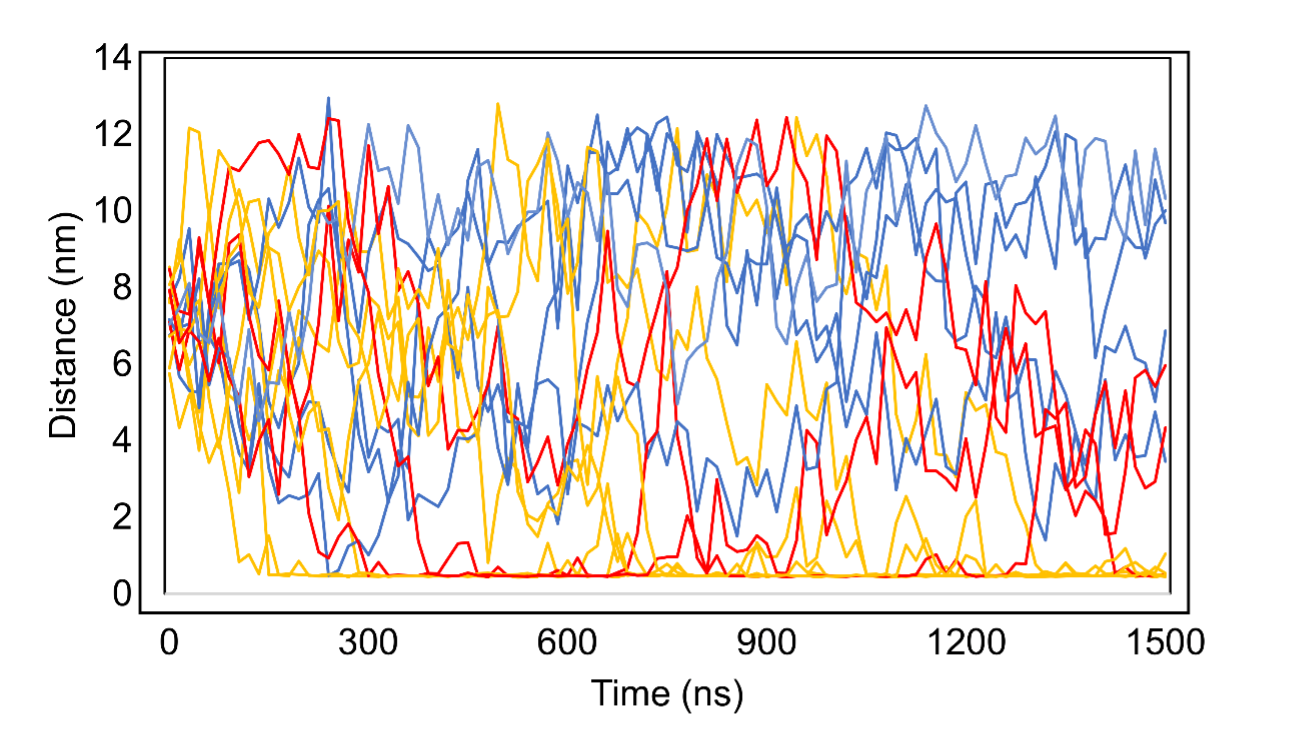

Supplement: S3 Fig — The minimum distance between the protein and bilayer is shown as a function of time for each of the 15 repeat simulations, showing no binding (blue, 7/15), non-canonical binding followed by dissociation (red 3/15), or non-canonical binding (yellow, 5/15). (TIF) [file pcbi.1008807.s003.tif]

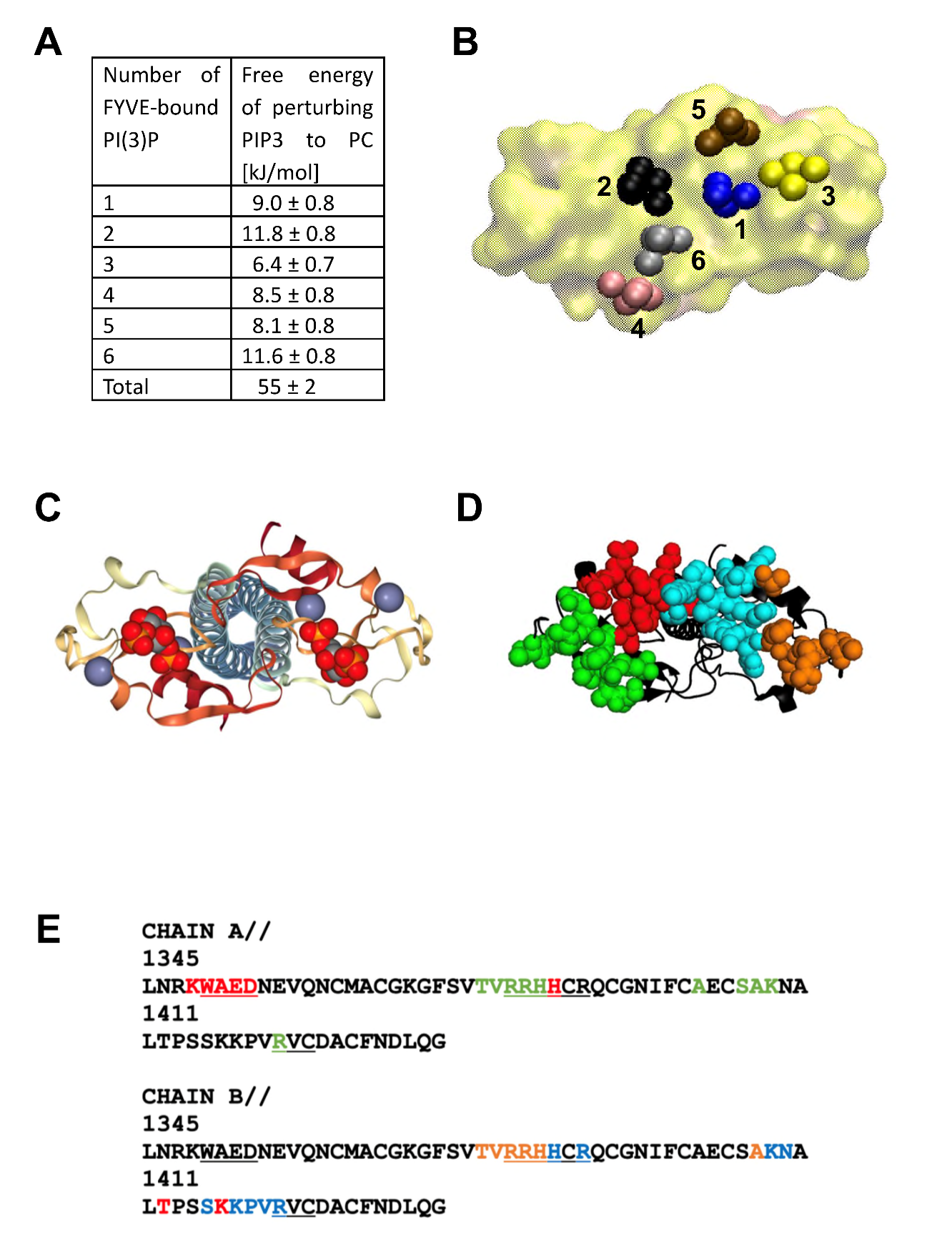

Supplement: S4 Fig — (A) Table of free energies for conversion of PI(3)P to PC at 6 selected sites (shown in (B) where the FYVE dimer, yellow, is viewed from the membrane facing side) where PI(3)P was seen bound in CG simulations. The table lists the corresponding FEP estimates for PI(3)P conversion to PC at each site. (C) Crystal structure (PDB ID 1JOC) of the FYVE dimer with two bound inositol (1,3)-biphosphates (red/orange/grey) and zinc ions (blue/purple spheres). (D) Binding sites on the FYVE dimer structure from CG simulations with the two most prominent binding sites on each monomer shown as coloured spheres. (E) Sequence of chains A and B from dimeric FYVE, highlighting the lipid contact sites using the same residue colours as in panel D, with conserved sequence motifs underlined. (TIF) [file pcbi.1008807.s004.tif]

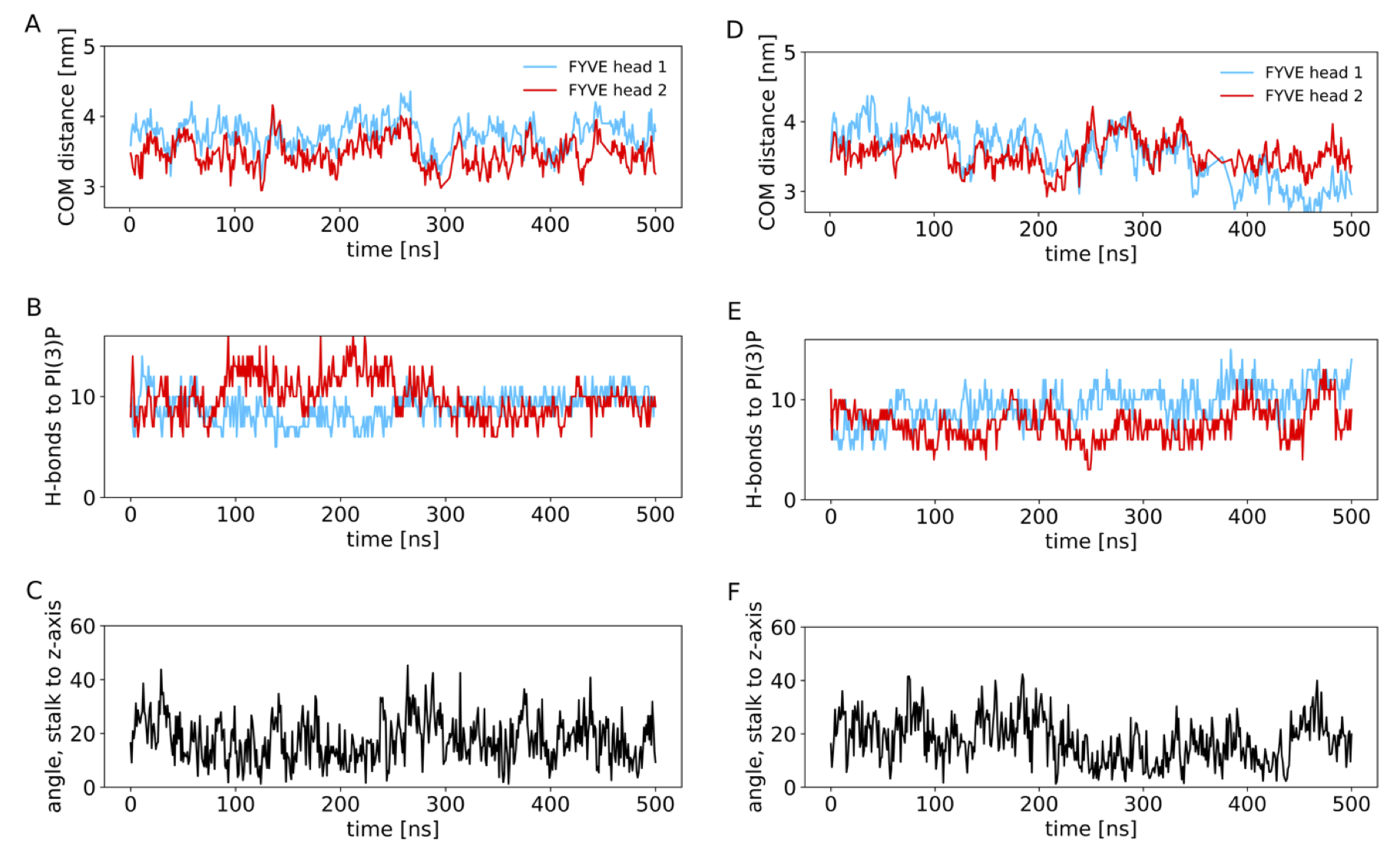

Supplement: S5 Fig — (A-C) repeat 2, (D-F) repeat 3. (A, D), centre of mass distance between each FYVE head and the membrane. (B, E) Number of H-bonds between each FYVE head and PI(3)P headgroups. (C, F) Angle between the coiled coil stalk axis and the membrane normal. (TIF) [file pcbi.1008807.s005.tif]

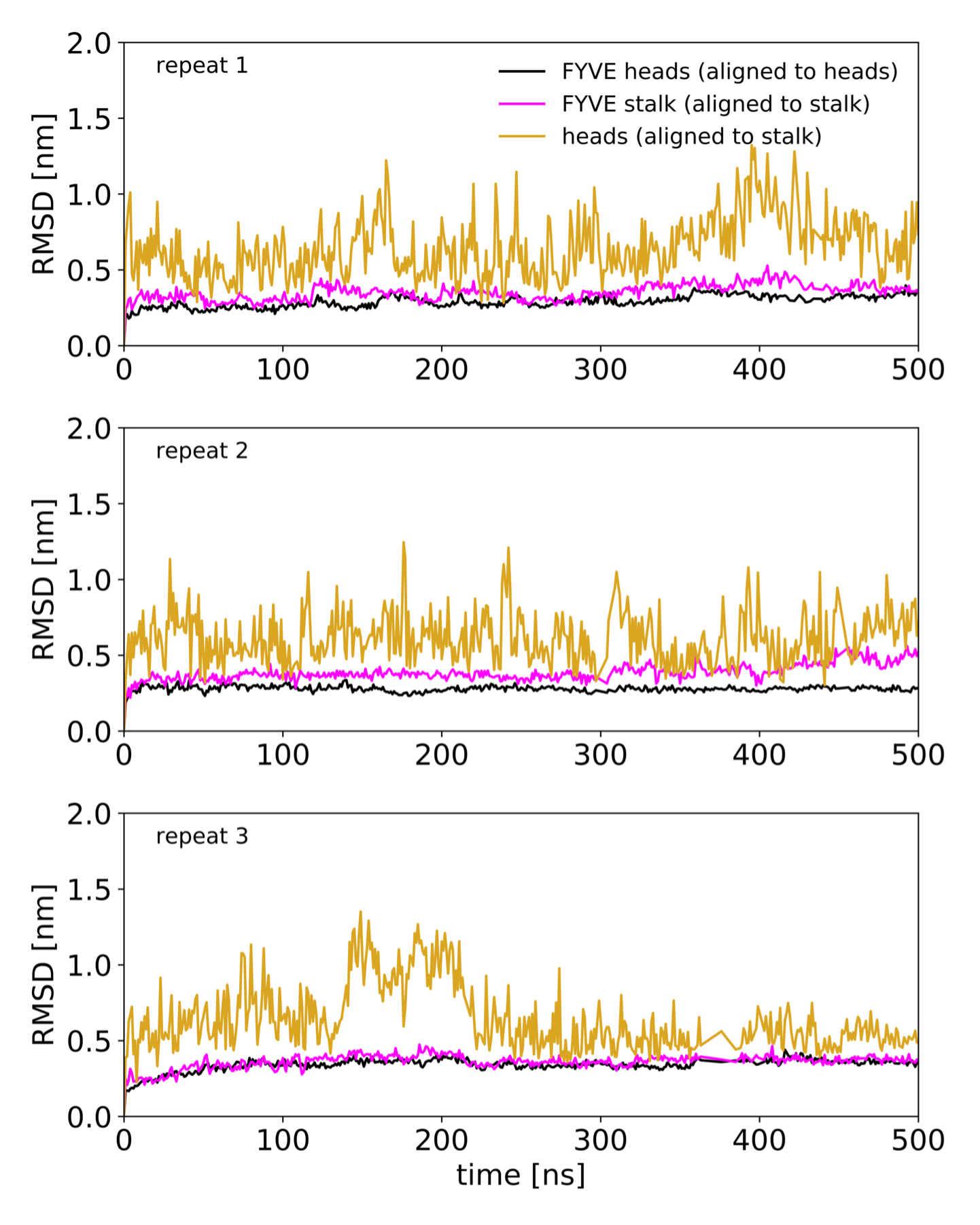

Supplement: S6 Fig — Cα RMSDs from the atomistic simulations are shown for the FYVE dimer heads aligned to the initial structure of the heads (black), for the coiled-coil stalk aligned to the stalk (purple), and for the heads with the dimer structures aligned to the stalk (gold). This demonstrates the rigid body motions between stalk and heads observed during the simulation. (TIF) [file pcbi.1008807.s006.tif]
