# Supplementary material for: Membrane-binding mechanism of the EEA1 FYVE domain revealed by multi-scale molecular dynamics simulations
Source: PLoS Comput Biol. 2021 Sep 23;17(9):e1008807. doi: 10.1371/journal.pcbi.1008807 (PMC8491906; doi:10.1371/journal.pcbi.1008807)
Supplement: Supplementary file 2 [file pcbi.1008807.s007.docx]

The paper describes an interesting attempt to simulate the binding of the EEA1 FYVE domain binds to endosomal membranes. The simulations are done by multiscale approach and seems to give reasonable results.
I only have a few comments.

1)I am not sure about the assessment of the dimerization effect. This should involve calculations of the dimerization free energy. This issue should be addressed.

2) Other approaches for CG and MD simulations of insertion of membrane proteins into membranes should have been mentioned and discussed. Including , Roux , Warshel (CG) , Ben-tal, Lindahl and others
