# Supplementary material for: Membrane-binding mechanism of the EEA1 FYVE domain revealed by multi-scale molecular dynamics simulations
Source: PLoS Comput Biol. 2021 Sep 23;17(9):e1008807. doi: 10.1371/journal.pcbi.1008807 (PMC8491906; doi:10.1371/journal.pcbi.1008807)
Supplement: Attachment — Submitted filename: responses v7.docx [file pcbi.1008807.s008.docx]

**PCOMPBIOL-D-21-00195 – Responses to Reviewers’ Comments**

***Reviewer #1:***

The manuscript by Sansom and co-workers describes atomistic and coarse-grained (CG) simulations of the C-terminal FYVE domain of EEA1 in the presence of membranes mimicking an endosome. The subject is of high biological interest, as the protein is involved in several diseases. The methodology is well-established and appropriate for the problem at hand. The manuscript is very clear in all parts. The general conclusions are supported by the data. I very much enjoyed reading the manuscript, however I would like to indicate a few points that should be improved:

- *We thank the reviewer for their very supportive comments and have revised the manuscript as described below.*

1. On calculating the protein-lipid binding affinity from a PMF: the correct way to do it was established by Shoup and Szabo (Biophys. J. 40, 33–39 (1982)), and has been reported by several others (see, for example, eq. 44 in DOI:10.1002/jcc.21776). Using the correct equations, the results will probably be similar - most likely this will not change dramatically the results.

- *This is true and we really should have done this in the first version. We have modified both the Result (page 5) and the Methods (page 9) accordingly.*

2. Page 6: "As the coiled coil in the full-length EEA1 protein is more than 200 nm long, and will be locally rigid, a tilt of up to ~50° relative to the bilayer normal would result in a later displacement of the Rab5 binding domain at the opposite end of nearly 2000 nm". I cannot follow the math. It seems to me that, if the stalk is about 200 nm in length, and Rab5 is attached to the opposite end of the FYVE heads, tilting the stalk by whatever amount would move Rab5 by 200 nm at maximum (provided that the stalk does not go through the membrane, does not unfold, and behaves as a rigid body). I also do not follow the math for the reachable area, that seems to be pi*(200 nm * (sin theta))^2 if theta is small (i.e., one can assume that the searchable area is a circle). If theta is large, the searchable area should be represented as a spherical cap [ area = 2*pi*r^2(1 - cos theta) ]. In any case, with theta = 50 degrees, the result I get for the searchable area is much less than 0.12 um^2.

- *We apologise for the mistake in (the corresponding author’s) arithmetic. This has now been corrected on page 7 ( …. provide a search area of at least 0.09 µm^2^…) and Fig. 5.*

Other points:

- On protein-lipid binding modes explored in AA simulations: it is apparent that the sampling achieved in the AA simulations is insufficient to assess the binding modes in a quantitative way. That is expected, considering the kinetics of protein binding/unbinding. I wonder if differences in binding modes were observed also in CG simulations. In that case, extension of the CG simulations would allow a more quantitative assessment of the probability of the different binding modes. If instead the CG model is not accurate enough in this respect, it would be interesting to determine which features/ingredients are missing in the CG model to enable mimicking the AA binding modes (electrostatics or hydrogen bonding too primitive? Shape of the binding pockets too different/smooth compared to AA? Other reasons?). In general, it would be interesting to make a more quantitative comparison between AA and CG binding modes.

- *In part, we have tried to address the sampling limitations of the AA simulations by replicate simulations (see below). However, we agree that sampling is still a limiting factor for more quantitative analysis. We therefore completely agree with the reviewer that it will in future be important to analyse the extent to which the CG/Martini model does/does not capture the key aspects of atomistic interactions of peripheral membrane proteins with PIP-containing membranes.* *Some possible limitations of the Martini model have been addressed with the release of Martini 3 (which was published after the current manuscript was submitted). However, the new version of the CG force field will require careful evaluation using simpler and better documented test systems (e.g. PH domains) of the Martini 3 parameters for protein/PIP interactions. In particular, it will be important to test Martini 3 simulated interactions against both AA simulations and against multiple experimental datasets obtained from different techniques and research groups in order to be confident of the improvement in Martini 3 PIP parameters. This will clearly be an important area of future research. These considerations are now discussed in general terms on page 7 of the revised manuscript.*

- On the hinge-bending motion observed in AA simulations: it is very unlikely that AA simulations converged or explored all thermodynamically accessible states in such short amount of simulation time (although, of course, it is recognised that these are computationally very expensive simulations). At the same time, it is also unlikely that the CG model will reproduce such motion realistically - because it is difficult to calibrate the elastic network. Perhaps a word of caution on the AA results should warn the reader that the 50 degree estimate is just an educated guess.

- *Yes, this is clearly the case. In response to a comment by Rev #2 we have run an additional two repeats of the AA simulations and so we are able to examine the variation in angle between 3 replicates, albeit on a 500 ns timescale (see SI Fig. S5). We are therefore able to add a suitable caveat as suggested by the Reviewer (see page 6).*

***Reviewer #2:***

Larsen & Tata et al. present a computational study on how the FYVE domain of EEA1 binds to PI3P-containing membranes such as those in endosomes. The computational work is solid (I only have one comment regarding the atomistic simulation), and the paper is clearly organized.

- *We thank the reviewer for their positive comments. We changed the manuscript as described below by running additional AA simulations.*

Main comments:
- Was there only one atomistic simulation? I think the atomistic simulation should be repeated at least two more times (or more, depending on the results of the two additional simulation runs), as it is hard to draw conclusions from a single simulation.

- *We agree, having just a single AA simulation was a shortcoming of the initial submission. We have therefore run a further 2x 0.5 µs and this has enabled us to strengthen the analysis and interpretation in the manuscript (see e.g. first paragraph of page 6).*

- It would be great to see the 15 starting orientations for the coarse-grained simulations in a supplemental figure.

- *This is a great suggestion - these have now been added to the SI (see SI Fig. S1).*

- In the Methods: "This crystal structure is positively charged at its N-terminal region (i.e. the start of the coiled coil stalk, residues 1289-1322 of EEA1), which resulted in artefactual binding of the N-terminus to the anionic lipid bilayer membrane. Therefore, we used a truncated version of the protein with a shorter coiled-coil stalk consisting of residues 1323-1411."---This is a bit confusing; please clarify exactly how the simulated system differs from the crystal structure and from wildtype EEA1.

- *We apologise for a rather terse and confusing description of this.* *The wildtype EEA1 has a ~200 nm long coiled-coil stalk, running from residues 74 to 1348, followed by a FYVE domain (residues 1352-1411). The crystal structure of the dimer (1JOC) has a truncated coiled-coil stalk formed by residues 1289-1348. Initial simulations of this construct resulted in biologically unrealistic interactions of the cationic N-terminal region (1289-1322) of this stalk with the bilayer. We therefore further shortened the stalk in the simulations so that it started at residue 1323, which resulted in biologically realistic interactions with the bilayer via the FYVE domain rather than the stalk. This is now explained in the text in more detail on page 8.*

- I think it would be good to briefly comment on the more general biological significance of the findings in the conclusions and/or abstract.

- *This is a good point – we have added a sentence to the end of the abstract and a couple of sentences at the end of the conclusions (page 7).*

Minor comments:
- In the abstract, I would change "... total binding energy is ..." to "estimated total binding energy" or similar

- *This has been changed accordingly.*

***Reviewer #3:***

The paper describes an interesting attempt to simulate the binding of the EEA1 FYVE domain binds to endosomal membranes. The simulations are done by multiscale approach and seems to give reasonable results.
I only have a few comments.

1)I am not sure about the assessment of the dimerization effect. This should involve calculations of the dimerization free energy. This issue should be addressed.

- *It would be of great interest to calculate the dimerization free energy for FYVE. However a key element of this is the stabilization of the dimer by the coiled coil stalk. Without this region, the FYVE domain on its own appears to be mainly monomeric, as seen in the NMR structure (e.g. PDB id 1HYI) of the stalk-less construct. To calculate the free energy of dimerization in the presence of the stalk is rather challenging for two reasons. Firstly, this would involve non-trivial definition of a suitable reaction coordinate, as the coiled-coil would need to be uncoiled. This could perhaps be achieved by a suitable collective variable within a metadynamics based approach. The second challenge is more pragmatic, requiring analysis of the free energy of dimerization as a function of different fragment lengths of the ~200 nm long coiled coil of EAA1. For these reasons we would wish to defer this very interesting suggestion for a future systematic study which would focus on the structure/stability of EAA1 per se. This is now mentioned briefly on page 5.*

2) Other approaches for CG and MD simulations of insertion of membrane proteins into membranes should have been mentioned and discussed.
Including , Roux , Warshel (CG) , Ben-tal, Lindahl and others

- *This is entirely correct. We apologise for the omission. We have provided a mention of some key such approaches for improved sampling of membrane/protein interactions and included references to key methods for these simulations on page 3 of the revised manuscript.*

***Reviewer #4:***

This manuscript describes atomistic (AT) and coarse-grained (CG) simulations aimed at understanding how the protein EEA1, specifically its C-terminal FYVE domain, binds to PI-containing membranes. From the CG simulations, they find that binding occurs via a hinge mechanism with a total free-energy gain of 67 kJ/mol, in reasonable agreement with experiments. AT simulations of the bound-state resolves binding into two types as well as demonstrates significant flexibility of the stem domain.

I found this manuscript very clear and well reasoned. My only suggestion is to include some snapshots in the SI of the non-canonical binding events. Currently, only a graph of distance is shown in Figure S1.

- *We thank the reviewer for their comments. This is a good suggestion and we have added a new SI figure (Fig. S2) to show these. This is mentioned on page 4 of the revised text.*

Minor points

Page 3: "was identified determined by" - remove one identified/determined

- *We have removed “determined”.*

Page 6: "head one bind" – binds

- *This text has been modified.*

Figure 4C: the time axis is incorrect; it's stated to be ns but is actually microseconds.

- *Our apologies – we have corrected this.*

**Have all data underlying the figures and results presented in the manuscript been provided?**
Large-scale datasets should be made available via a public repository as described in the *PLOS Computational Biology* [data availability policy](http://journals.plos.org/ploscompbiol/s/data-availability), and numerical data that underlies graphs or summary statistics should be provided in spreadsheet form as supporting information.

- *We now provide a link to scripts and input files in the Methods. Unfortunately, there is still not an accepted public repository for MD simulations.*
