# Supplementary material for: Membrane-binding mechanism of the EEA1 FYVE domain revealed by multi-scale molecular dynamics simulations
Source: PLoS Comput Biol. 2021 Sep 23;17(9):e1008807. doi: 10.1371/journal.pcbi.1008807 (PMC8491906; doi:10.1371/journal.pcbi.1008807)
Supplement: Attachment — Submitted filename: Data deposition.docx [file pcbi.1008807.s009.docx]

**Data deposition**

We have deposited the following data:

1. Scripts etc for the simulations are uploaded to GitHub:

<https://github.com/andreashlarsen/Larsen-Tata2021-FYVE>

with associated DOI: 10.5281/zenodo.5048289

2. MD simulation trajectories are deposited at Zenodo:

<https://zenodo.org/record/5362218#.YTDSpS0Rpqs>

with DOI: 10.5281/zenodo.5362218

This entry includes a overview of the files deposited.

The above information is now included in the manuscript, final paragraph, page 12.
